# Mapping drug-target interactions and synergy in multi-molecular therapeutics for pressure-overload cardiac hypertrophy

**DOI:** 10.1038/s41540-021-00171-z

**Published:** 2021-02-15

**Authors:** Aparna Rai, Vikas Kumar, Gaurav Jerath, C. C. Kartha, Vibin Ramakrishnan

**Affiliations:** 1 Molecular Informatics and Design Laboratory, Department of Biosciences and Bioengineering, Indian Institute of Technology Guwahati, Guwahati, Assam India; 2 Laboratory of Cardiovascular Science, National Institute on Aging, National Institutes of Health, Baltimore, MD USA; 3 PepThera Laboratories Private Limited, Guwahati, Assam India; 4 Society for Continuing Medical Education & Research, Kerala Institute of Medical Sciences, Thiruvananthapuram, Kerala India

**Keywords:** Target identification, Clinical pharmacology, Biochemical networks, Cardiology, Cheminformatics

## Abstract

Advancements in systems biology have resulted in the development of network pharmacology, leading to a paradigm shift from “one-target, one-drug” to “target-network, multi-component therapeutics”. We employ a chimeric approach involving in-vivo assays, gene expression analysis, cheminformatics, and network biology to deduce the regulatory actions of a multi-constituent Ayurvedic concoction, Amalaki Rasayana (AR) in animal models for its effect in pressure-overload cardiac hypertrophy. The proteomics analysis of in-vivo assays for Aorta Constricted and Biologically Aged rat models identify proteins expressed under each condition. Network analysis mapping protein–protein interactions and synergistic actions of AR using multi-component networks reveal drug targets such as ACADM, COX4I1, COX6B1, HBB, MYH14, and SLC25A4, as potential pharmacological co-targets for cardiac hypertrophy. Further, five out of eighteen AR constituents potentially target these proteins. We propose a distinct prospective strategy for the discovery of network pharmacological therapies and repositioning of existing drug molecules for treating pressure-overload cardiac hypertrophy.

## Introduction

Modern medicine is primarily driven by the discovery of small-molecule entities with pharmacological actions^1^. Despite the vast scope of the chemical universe, the probability of success in discovering a new molecule with potential therapeutic effects is now becoming more and more challenging. In addition, the “one drug, one target” mode of drug action cannot generally lead to multiple effects in complex or multifactorial diseases because of the underlying complexity of biological networks^2–5^. The disease is known to be a rare consequence of an abnormality in a single gene or gene product but reflects the perturbations of a complex intracellular and intercellular network in organ systems. The emerging tools of network medicine offer a platform to explore not only the molecular complexity of a particular disease, leading to the identification of disease modules and pathways, but also in investigating molecular defects among apparently distinct pathological phenotypes^6–8^. A sensible approach for the treatment of complex diseases is to have a combinatorial drug with constituents that target multiple pathways in a disease-specific network^9,10^.

The complexity of the underlying biological processes and interactome, therefore, demands multiple synergistic therapeutics targeting different proteins involved in the disease onset and prognosis. Identification of such synergistic therapeutic partners is both complex and arduous due to various drug–drug interactions. We believe that it would be prudent to identify traditional medicines in eastern methods, which have been practiced over centuries, for their potential utility^11^. To bring such therapeutic solutions to the platform, modern medicine can be a rewarding exercise. As a test case, we have identified Amalaki Rasayana (AR), a concoction used for the treatment of cardiovascular diseases, diabetes, and rejuvenation therapies in Ayurveda, with an objective to re-invent its efficacy through established analytical procedures of modern medicine.

Cardiac hypertrophy due to pressure overload or pressure-overload left ventricular cardiac hypertrophy (LVCH), is one of the leading causes of mortality in recent times, especially in the younger population and patients having hypertension^12,13^. The physiopathological characterization of hypertrophy is multifactorial and it involves a higher degree of complexity at the cellular and molecular level, across multiple signaling pathways^14^. Pressure-overload LVCH is a significant phenomenon in cardiac hypertrophy and accounts for 10–15% cases in the adult population and ~40% with hypertension, leading to adverse cardiovascular events such as heart failure and sudden death^15^. Therapeutic interventions such as the use of ACE and myosin inhibitors and various anti-hypertensive drugs have significant clinical benefits but have limited success due to the ceiling effect in many patients^16,17^. Therefore, extensive research on the molecular basis of this complex, multifaceted physiopathological malady at the levels of pathways, drug dosage, and targeted therapy can be helpful in proposing new therapeutic options^18,19^.

Interestingly, Ayurveda, the ancient Indian system of medicine employs combinatorial therapy^11,20^. Ayurvedic medicines have been refined and evolved continuously over centuries and is one of the most dependent forms of alternative medicine for more than 1.5 billion population living in the Indian sub-continent. Ayurvedic medicines commonly have multiple components derived from natural sources and are believed to work through a cooperative mechanism or “synergy” to restore the balance of life and body functions through heightened physiological response, not achievable by individual constituents^21,22^. Synergistic action maybe because of the involvement of a set of targeted proteins, related mechanisms, or drug combinations. In modern drugs, synergistic action is due to their combinatorial effects on multiple targets identified by their network topological features. Synergistic action predictions are generally pathway-based, drug similarity-based, and using omics-based models^23–26^. Advancement in systems biology has resulted in a new concept known as “network pharmacology”, which offers a radical shift from the existing paradigm of “one-target, one-drug” mode to a “network-target, multiple-component-therapeutics”^27^. We present a unique approach, integrating the tools of cheminformatics, proteomics, in-vivo experiments, and network analysis to identify biological targets and synergy of constituents in a typical multi-molecular medicinal formulation for complex diseases. We analyzed the cumulative in-vivo effect of AR, a concoction used by practitioners of Ayurveda for centuries for the treatment of pressure-overload LVCH^28^. The data generated through in-vivo studies were utilized to detail the possible drug targets for each of the constituent metabolites of AR. We also identified those AR metabolites, structurally similar to known drugs and potential targets in modern medicine. Further, we also identified a set of existing drug molecules that could be re-purposed for pressure-overload LVCH.

## Results

### in-vivo studies for AR efficacy

**Fig. 1:**
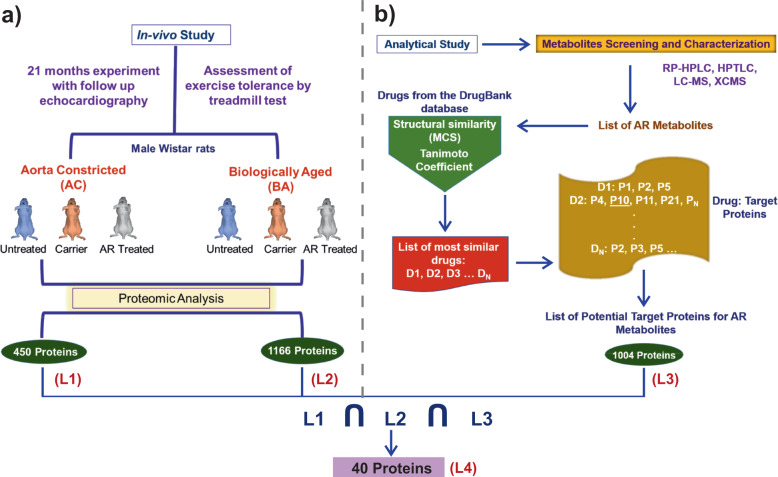
in-vivo studies and data extraction. **a** Illustration presenting an overall schematic representation of the in-vivo study performed on male Wistar rats for 21 months^28^. The proteins from the AC and BA samples were identified by proteomic analysis and designated as L1 and L2, respectively. **b** The AR composition was identified by mass spectroscopy techniques. The AR constituent metabolites, thus identified were used to search structurally similar drugs in the DrugBank database. Further, the targets corresponding to the highest-ranked drug molecules were identified to relate their activity against each metabolite (L3). The intersection of L1, L2, and L3 had 40 proteins (L4). These proteins represent the set of proteins targeted by various AR metabolites, which leads to the drug action of the AR concoction. Therefore, we used list L4 for our further analysis.

AR is a result of a series of systematic preparatory procedures, prepared primarily from the fruits of *Phyllanthus Emblica or Emblica Officinalis* (as major constituent) as per the guidelines of Charaka Samhita, written in the third century BCE^29^. In an earlier experiment, spanning a total period of 21 months, we considered two groups of Wistar rats; (i) Aorta Constricted (AC) with pressure-overload LVCH, induced by clipping ascending Aorta with titanium clips, and (ii) Biologically Aged (BA) rats (Fig. 1a). Both these groups individually were further sub-grouped, where these animals were either given AR (AR treated, Test sub-group) or ghee and honey treated (carrier sub-group) orally by guavage regularly. Another sub-group in both the AC and BA groups acted as control (Untreated sub-group), where no drug was administered to the animals^28^. Thereafter, we evaluated the effects of long-term administration of AR on the structure and function of the heart. Histology, as well as gene and protein expression analysis, were done in left ventricular heart tissues, collected after the sacrifice of the animals. In the 21-months experiment on Wistar rats, we found that AR intake resulted in an improved left ventricular function and decreased left ventricular hypertrophy in AC rats. AC groups fed AR also had increased fatigue time in the treadmill exercise test. BA group fed AR also had improved left ventricular function. Further, the protein expression profile of the heart tissue of both AR and BA groups with AR intake revealed upregulation of SERCA2, CaM, Myh11 as well as antioxidant, autophagy, oxidative phosphorylation, and tricarboxylic acid (TCA) cycle proteins. ADRB1/2 and pCREB expressions were also increased, but the expressions of pAMPK and NF-kB were decreased. The protein expression analysis indicates that AR has a beneficial effect on myocardial energetics, muscle contractile function, and exercise tolerance capacity in rats^28^. We identified 450 proteins (List L1) in cardiac tissues of AC rats, and 1166 proteins in cardiac tissues of BA rats (List L2). The Lists L1 and L2 along with their expression profiles are given in the Supplementary Dataset S1.

In addition to the above experiment, AR was characterized by a chemical composition analysis using HPLC, HPTLC, and LC-MS which yielded 18 constituent Metabolites (Table 1). These constituents were found to have biological importance in the cardiovascular system. The HPTLC profiles of samples of the finished formulation indicate the presence of gallic acid and ellagic acid (Supplementary Fig. S1). The LC-MS analysis of lyophilized powder of AR revealed the enrichment of components such as putative anti-inflammatory arachidonate (eicosatetraenoic acid), norepinephrine sulfate, and vitamin metabolites, identified using XCMS software used for metabolomics study^30^ (Supplementary Tables S1 and S2).

**Table 1:** AR constituent metabolites.

Serial no.	Metabolite	Associated pathways/functions
1.	Gallic acid	Mucous protection, astringent effects
2.	Ellagic acid	Antioxidant and anti-proliferative effects
3.	Biocytin	Vitamin H (biotin) metabolism
4.	Methylcobalamin	Vitamin B12 (cyanocobalamin) metabolism
5.	L-methionine	Amino acid metabolism
6.	Pyridoxamine phosphate	Vitamin B6 (pyridoxine) metabolism
7.	Prostaglandin B1	Prostaglandin formation from arachidonate
8.	4-Hydroxy-all-trans-retinyl_acetate	Vitamin A (retinol) metabolism
9.	13’-carboxy-alpha-tocotrienol	Vitamin E metabolism
10.	Cholic acid	Bile acid metabolism
11.	1alpha,24 R,25-trihydroxyvitamin D3 (calcitetrol)	Vitamin D3 (cholecalciferol) metabolism
12.	Guanylic acid	Purine metabolism
13.	Carbamoyl phosphate	Pyrimidine metabolism
14.	5-formiminotetrahydrofolate	Vitamin B9 (folate) metabolism, Histidine metabolism
15.	Nicotinate D-ribonucleoside	Vitamin B3 (nicotinate and nicotinamide) metabolism
16.	Sulfate derivative of norepinephrine (noradrenaline sulfate)	Tyrosine metabolism
17.	12-oxo-20-dihydroxy-leukotriene_B4	Leukotriene metabolism
18.	5S,6S-epoxy-15S-hydroxy-7E,9E,11Z,13E-eicosatetraenoic acid	Arachidonic acid metabolism or putative anti-inflammatory metabolite

The list of AR constituent metabolites identified from HPLC, HPTLC, and LC-MS analysis.

### Drug–metabolite similarity search

The cheminformatics analysis in this study was firmly grounded on the basic idea that similar structures, with chemically identical functional groups, tend to possess similar functions^31–33^. Considering the list of AR metabolites identified earlier (Table 1)^28^, we screened the DrugBank database with 9000 approved and experimental drugs to identify molecules that are structurally similar to AR metabolites^34^. We compared the two-dimensional structure of drugs with those of AR metabolites using fragment-based drug discovery. Our approach includes the identification of maximum common substructure (MCS) between the metabolites and known drug molecules through local and global structural similarity search using the Tanimoto Coefficient (Tc) (Fig. 1b)^35^. We used a Tc cutoff of 0.6 for quantifying the structural similarity between two molecules. The screened drug molecules were further verified manually by visualizing the pairwise similarity between drug molecules and the AR metabolites using Small Molecular Subgraph Detector (SMSD) toolkit^36^. The list of AR metabolites and drugs screened are given in the Supplementary Dataset S1. Following the basic paradigm of a similar structure can lead to similar mechanisms of action; targets of structurally similar drugs can also be the targets for the AR metabolites. We thus obtained a list of AR metabolites and their associated potential target proteins (L3). Altogether, 1004 targets were identified (Supplementary Dataset S1). This list includes a set of targets of known drugs, which can be potentially targeted by the AR metabolites as well.

### Protein–protein interaction network analysis screen important proteins

**Fig. 2:**
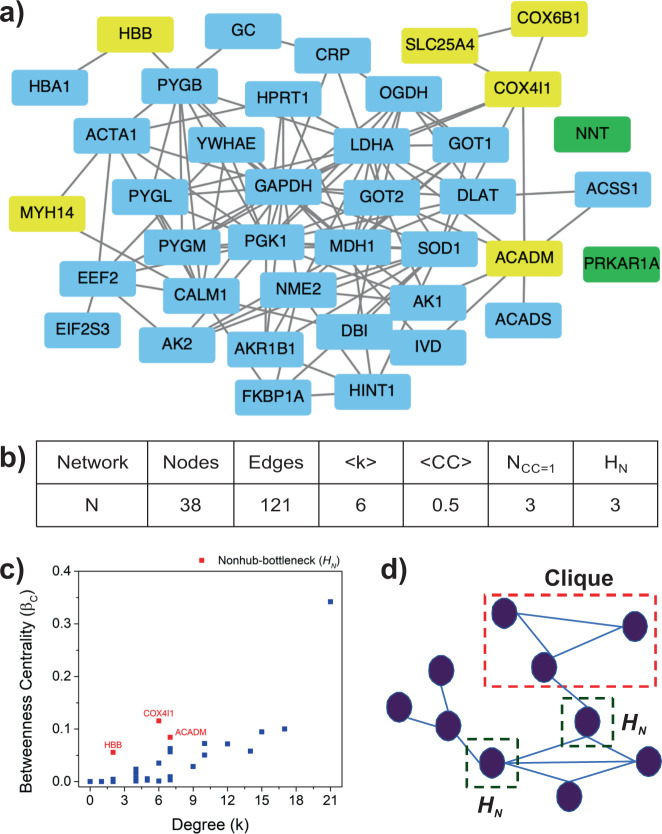
PPI network. Using STRING database, PPI network for 40 common proteins was constructed. **a** The network has 38 nodes and 121 edges. Nodes namely PRKAR1A and NNT (Green Boxes) do not interact with any of the other nodes. For statistical analysis, the largest connected component was considered and the unconnected nodes were ignored. The yellow boxes are the set of targets obtained from network analysis which were further studied for their therapeutic potential against cardiovascular diseases. **b** The table illustrates various structural properties of the network corresponding to 40 common proteins, where *N* refers to the name of the network followed by the number of nodes and edges in the network. <*k>* represents the average degree of the network along with the average clustering coefficient (*<CC*>). *N*_*CC=1*_ is the number of nodes having *CC=1*, whereas *H*_*N*_ represents the nonhub bottlenecks that have a very low degree but high betweenness centrality. **c** The betweenness centrality *Vs* degree curve enlisting nonhub bottlenecks (*H*_*N*_) as red dots in the graph. **d** Schematic representation of clique structures (red dotted rectangle) and *H*_*N*_ (green dotted square) property in a network. Cliques are closed subgraphs in a network that are vital to the network stability and robustness. *H*_*N*_ represents the nodes that act as bridges between consecutive network components like hub nodes and subgraphs. Targeting such nodes helps to maintain network integrity and, therefore, is hypothesized to be ideal pharmacological drug targets in the network.

Network biology has been widely used in understanding the complexity of various diseases, their interacting patterns, role, and the importance of interaction patterns^37–41^. Lists L1 and L2 represent the proteins, whose expression was perturbed due to the administration of AR. The L3 list described above-provided information on potential drug targets that can also interact with the AR metabolites. Therefore, the intersection of the three lists (L1, L2, and L3) would represent the list of proteins responsible for the disease prognosis, which when targeted through AR metabolites lead to the therapeutic action. The intersection of the three lists (L4) has 40 proteins (Supplementary Dataset S1). Next, we constructed the protein–protein interaction network of the L4 proteins and analyzed the network for various topological properties (Fig. 2a), with an aim to identify the proteins responsible for network/pathway integrity.

The connected component of the PPI network (N) constructed using L4 proteins comprised of 38 nodes (proteins) and 121 edges (interactions). The interaction data of the proteins in the form of an adjacency list can be found in Supplementary Dataset S1. The p-value of the PPI enrichment according to the STRING database was 1.0e-16, which indicated that the interaction data are statistically significant. Such an enrichment score suggested that proteins have more interactions among themselves than what would be expected for a random set of proteins of similar size and are biologically significant as a group. To get a deeper insight, the network was analyzed for various structural/topological properties listed in Fig. 2b and Supplementary Table S3. The fundamental property, average degree, *<k>* of the network was 6, indicating interconnectedness of the nodes in the network. The degree of fourteen nodes higher than the average degree in the network suggests their function in promoting the network’s robustness through their connectedness against random external perturbations^42,43^. This was further confirmed by pathway analysis using the STRING database, where the cutoff for false discovery rate (FDR) was <0.05 (95% significant). These results were further verified from KEGG Mapper, Gorilla, and DAVID databases^44–47^. The ontology from the STRING database indicated that 28 out of 38 proteins are involved in metabolic pathways and 10 of them were found in carbohydrates metabolism. The detailed ontology of the proteins has been enlisted in the Supplementary Table S4.

Next, the average clustering coefficient *(<CC>)* of the network N was 0.5, signifying intermediate connectivity among the neighbors of the nodes and the presence of complete subgraphs or clique structures^48,49^. There were three nodes with clustering coefficient *(CC)* as one i.e., all neighbors connected to each other, forming cliques. Cliques are essential as they are building blocks and the backbone of the network^50^. Clique structures in the network make the network robust^51^ and stable^52^ as they are also involved in the evolutionary process of the network from one stage to the other^53,54^. The three proteins having *CC* = *1* were COX6B1, MYH14, and SLC25A4 (Table 2). The proteins were further studied for their biological function through available literature, revealed their involvement in cardiac muscle contraction, neuropathy, myopathy, and oxidative phosphorylation. The protein COX6B1 is involved in cardiac muscle contraction^55^. This protein encodes an integral, nuclear-encoded COX subunit^55^. Mutation in the gene corresponding to COX6B1 protein, where histidine is replaced by cysteine at R20 residue, leads to reduced expression of COX6B1 protein in muscle and fibroblasts causing hypertrophic cardiomyopathy or cardiac muscle dysfunction^55^. An autosomal dominant mutation in the protein MYH14 leads to neuropathy, myopathy, hoarseness, and hearing loss^56,57^. SLC25A4 is active in cardiac hypertrophy and myopathy after mutation^58^. It is also reported that SLC25A4 controls oxidative phosphorylation, specifically to regulate energy phosphate levels associated with low ATP demand. This is directly associated with cardiac hypertrophy^58^. These proteins are having a vital position in the network, vital in disease prognosis, can therefore be suggested as a probable drug targets.

**Table 2:** Potential network targets.

Serial no.	Protein	Metabolite	Similar drugs
1.	ACADM	Guanylic acid	DB03147
2.	COX4I1	Cholic acid, L-methionine	DB02659, DB04464
3.	COX6B1	Cholic acid, L-methionine	DB02659, DB04464
4.	HBB	Gallic acid, cholic acid	DB08262, DB07645
5.	MYH14	Guanylic acid	DB03126
6.	SLC25A4	Guanylic acid	DB00171

The proteins identified as potential targets from the network analysis are listed. The corresponding AR metabolites targeting these proteins with chemically and structurally similar drugs (DrugBank accession IDs) are listed.

Further, the betweenness centrality *(β*_*c*_*)* of the nodes in the network signifies the participation of nodes in multiple pathways, indicating a positive correlation between the *β*_*c*_ of a node with its degree^59,60^. A node with a low degree and high betweenness centrality is termed as nonhub bottlenecks (*H*_*N*_*)*. This parameter is crucial as it detects the nodes with occurrence in the maximum number of pathways. The targeting of high degree nodes may collapse the whole system as they are maximally connected. Therefore, such nonhub bottlenecks (*H*_*N*_) are important for the network as they help to identify a weak breaking point of the network without compromising the whole system^61^. Three proteins ACADM, COX4I1, and HBB were identified in the nonhub bottleneck regime (Table 2), which are functionally involved in fatty acid metabolism, cardiac muscle contraction, and regulation of blood pressure. ACADM protein is associated with fatty acid metabolism^62^. Fatty acids and associated lipids are important determinants of both structure and function of cardiomyocytes. There is considerable evidence that in the postnatal and adult mammalian heart, fatty acid β oxidation is the preferred pathway for the energy that is required for efficient cardiac pumping^62,63^. A major switch in the myocardial bio-energetic substrate used, from fatty acid to glucose leads to downregulation of fatty acid β enzyme, leading to cardiac failure^55^. COX4I1 protein such as COX6B1 has a significant role in cardiac muscle contraction and its defect can result in heart failure, whereas HBB is responsible for the regulation of blood pressure^55^. The COX4I1 protein, in case of myocardial insufficiency (heart failure) and dilated cardiomyopathy, is found to have decreased expression of COX4I1, which results in an impaired cytochrome *c* oxidase (CytOx) activity that has an important role in myocardial respiration, ultimately affecting the mitochondrial respiratory chain^64,65^. Higher enzymatic activity but equal oxygen consumption contribute to the pathophysiology of myocardial insufficiency, which is an indicator of oxidative stress. The HBB protein has a vital role in the iron (Fe^2+^) binding and oxygen-binding during the transport of blood in the circulatory system, which regulates blood pressure and blood vessel diameter in the body^66^. Thus, these proteins can have therapeutic potential in treating cardiovascular diseases.

### Combinatorial effects of the AR metabolites

**Fig. 3:**
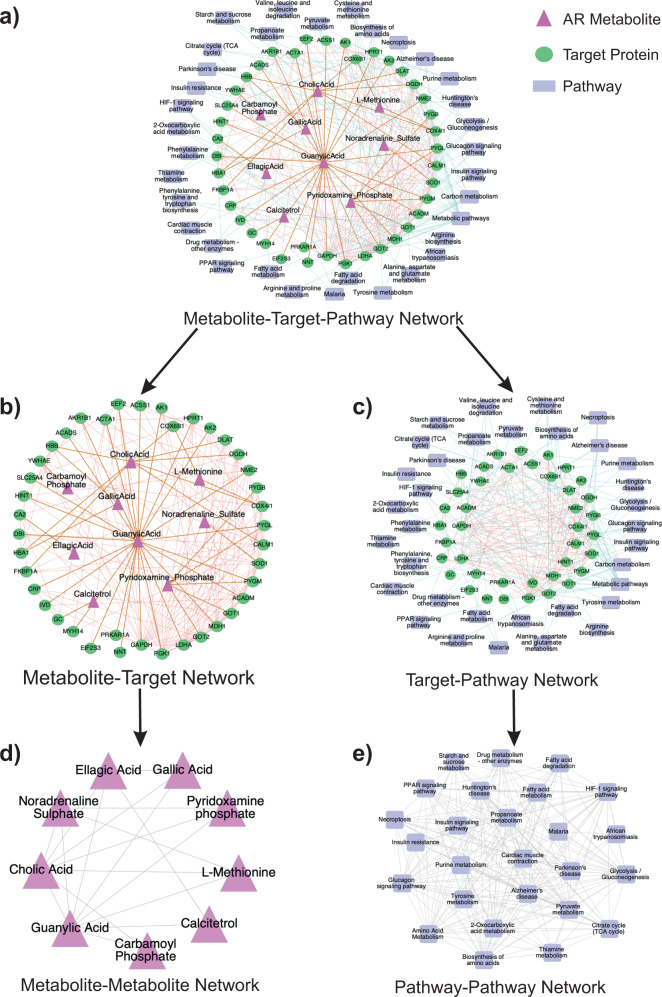
Synergistic action of AR metabolites. **a** Metabolite-Target-Pathway (MTP) network was constructed by connecting metabolites with their corresponding target proteins in the protein–protein interaction (PPI) network. The target proteins were further connected with their associated pathways to produce the final MTP network. The metabolites are shown as pink triangles, target proteins are represented as green circles, and pathways as blue rectangles. **b** The metabolite-target protein interaction network (MT network) is the subgraph of the MTP network and consists of the interaction of metabolites with their corresponding target proteins and interactions among the target proteins. **c** The target protein–pathway interaction network (TP network) consists of the protein–protein interactions and protein-pathway relations. The proteins are shown in green, while the pathways are shown in blue. **d** MT network was further reduced to a metabolite–metabolite interaction network (MM network) using a distance cutoff of ≤3 in the MT network for each metabolite pairs to identify a direct connection in the MM network. The direct association between two metabolites signifies their co-action in the regulation of single or multiple pathways. **e** TP network was reduced to a pathway–pathway interaction network (PP network). The PP network was constructed using the same principle used in the construction of the MM network. The adjacency (direct connection) of two nodes signifies the co-regulation of two pathways due to the action of Amalaki Rasayana metabolites.

The proteins described in the PPI network discussed above were associated with nine of the 18 AR constituent metabolites (Supplementary Table S5). The interconnectedness of the PPI network indicates that these metabolites have a synergistic action, which we analyzed through the guilt-by-association approach^4^. Multi-component or multilayered networks have been previously used for determining the synergistic drug action^4^. The basic principle involved in determining a synergistic action or synergistic partners is that two nodes of a type (metabolite/protein/pathway) should be connected through either a common or neighboring set of nodes of another kind. Therefore, to determine the synergy among AR metabolites, their target proteins, and the associated pathways; we constructed a multi-component network comprising AR metabolites, their potential protein targets, and associated pathways aka metabolite-target-pathway (MTP) network (Fig. 3a). This MTP network was used to construct two bi-layer networks, Metabolite-Target (MT) network and Target-Pathway (TP) network (Fig. 3b, c).

The synergy at the metabolite level was determined by constructing a subnetwork from the constructed MTP network using metabolites and target proteins only (MT network, Fig. 3b). For any pair of metabolites to be synergistic partners, they should target the same or directly connected targets in order to affect a single or set of related pathways. Therefore, using a path length cutoff of ≤3 between two metabolites in the MT network for a direct connection, we created a metabolite–metabolite (MM) network, wherein directly connected nodes are synergistic couples (Fig. 3d). The analysis revealed that guanylic acid was directly connected to all the remaining metabolites (eight neighbors), indicating that guanylic acid is the most active synergistic ingredient of AR followed by cholic acid (seven neighbors). The other metabolites had 2–4 direct neighbors, and the MM network was a closed graph. This suggests that all the AR metabolites are directly or indirectly synergistic partners, and present themselves as a collective network of medicine.

To analyze the synergistic effect of the related pathways in the pharmacological action of AR metabolites, we used the target-pathway (TP) part (Fig. 3c) of the MTP network. A network medicine essentially affects multiple pathways simultaneously (co-regulation of pathways), in contrast to the traditional one molecule-one target-one pathway mode of drug action^6,7^. Therefore, to study the co-regulation of different pathways by AR metabolites, we reduced the TP network to a pathway–pathway (PP) network, by using a path length cutoff of ≤3 in the TP network among pathway node sets to construct a direct connection between nodes in the PP network. The resulting PP network (Fig. 3e) is representative of different pathways co-regulated by the synergistic action of AR metabolites. PP network was observed to be a closed network comprising of 26 nodes (pathways) and 218 edges. The network being closed suggests high density which is evident from the average degree *<k>* of this network that comes out to be 17. Another topological feature namely diameter (D) for this network, was 2, indicating faster signaling^40^. Further, a very high average clustering coefficient (*<CC*>) of 0.9 signifies the presence of complete subgraphs, or in other words, it indicates that the pathways are interconnected. These network characteristics suggest that the co-regulation of all the discussed pathways in the PP network is critical to disease treatment and therefore, the removal of even one of the nine metabolites from the AR concoction could result in the disruption of pharmacological action, thereby, reaffirming that the AR metabolites have a synergistic response.

**Fig. 4:**
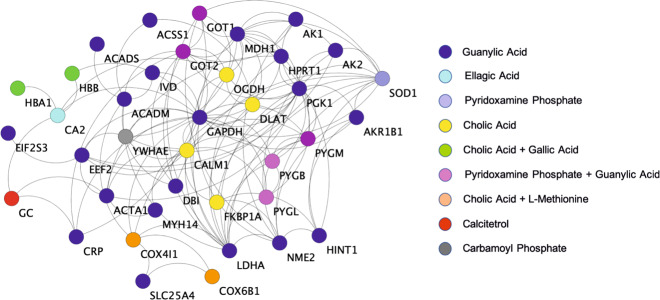
Synergistic action of AR metabolites on target proteins. The color codes on the proteins/targets in the interaction network indicate their corresponding metabolites identified after screening using SMSD and Tc. Blue circles represent the proteins corresponding to guanylic acid (the maximum in number), followed by yellow color for cholic acid. ellagic acid is represented by cyan color, whereas calciterol by red circles. Gray color circles correspond to carbamoyl phosphate and purple to pyridoxamine phosphate. Noradrenaline sulfate metabolite corresponds to light blue circled proteins. Few proteins correspond to more than one metabolite and are represented as orange circled proteins for the combination of cholic acid and L-methionine, and green circles for cholic acid and gallic acid. The pink circles are proteins corresponding to pyridoxamine phosphate and guanylic acid. All these proteins are interconnected and have a significant role in the disruption or regulation of pathways as seen when knocked out from the network. This observation suggests the co-dependency of AR metabolites for the maintenance of the protein–protein interaction network, which is vital to drug action, thereby affirming their synergistic action.

To understand the synergy of drug action at the target level, we investigated the protein–protein interaction network. The synergistic effect of metabolites in this PPI network was studied through the interconnectedness among the target proteins of different metabolites. The PPI network created is part of a larger disease-specific PPI network, which is targeted by the metabolites. Therefore, the network (Fig. 4) is vital for disease treatment and the collapse of this network would lead to a disruption of drug action. The knockout or deletion of individual metabolite-associated target proteins in the network will disrupt the whole network. Thus, we categorized the proteins in accordance with their corresponding metabolites (Fig. 4). We observed that various proteins associated with different metabolites are vital for maintaining network integration. This was evident from the proteins associated with guanylic acid, as they are maximally present in the underlined network, further suggesting that this metabolite has significant involvement in response to disease treatment. The other metabolites such as cholic acid, gallic acid, ellagic acid, etc. associated proteins provide a bridge of connections for the connectivity of guanylic acid-associated proteins among each other and in other pathways. This observation signifies the utility of multiple bridging metabolites for the therapeutic action of guanylic acid. This behavior was analyzed by studying the ontology of these proteins that revealed the interrelation of the proteins in the regulation of multiple pathways, such as metabolic pathways, glycolysis/gluconeogenesis, insulin signaling pathway, fatty acid metabolism, cardiac muscle contraction, etc. (Supplementary Table S4).

The three levels of synergistic action investigated, thus, reveal that the action of AR constituents is combinatorial and reflective of the modern concept of network medicine.

### Drug repositioning for pressure-overload LVCH

The six proteins ACADM, COX4I1, HBB, COX6B1, MYH14, and SLC25A4 correspond to four metabolites namely guanylic acid, cholic acid, L-methionine, and gallic acid. These metabolites may have therapeutic effects in the treatment of pressure-overload LVCH. Therefore, we studied the biological functions of these candidate metabolites for therapeutic implications and verified their binding affinity with the target proteins. Guanylic acid is an experimental drug and mechanisms in which this metabolite involved are unknown. This metabolite belongs to a class of organic compound ribonucleoside 3’-phosphates that contain a phosphate group attached to the C-3 carbon of the ribose or deoxyribose unit. It is known to be involved in uracil phosphoribosyltransferase activity^67^. Cholic acid is an approved drug that has a major role in primary bile acid production in the liver. It facilitates fat absorption and cholesterol excretion. This metabolite is primarily used in the treatment of children and adults with bile acid synthesis disorders and for peroxisomal disorders (such as Zellweger syndrome—bile enzyme malfunction)^68,69^. L-methionine is an approved drug and nutraceutical that helps to lower cholesterol levels by increasing the production of lecithin in the liver^34^. It reduces liver fat, protects the kidneys, and prevents disorders relating to hair, skin, and nails. The metabolite is a sulfur-based essential amino acid that acts as a natural chelating agent for heavy metals and also regulates the production of ammonia in urine^70^. Gallic acid is an approved drug that possesses protective effects on the gastric mucosa and has strong astringent effects. It is used as a deodorizing agent in medicines. The metabolite plays a major role in forming a protective coat on the intestinal mucosa and treating ulcers e.g., ulcers from *H. pylori*^71^. It has antimicrobial effects against various gastrointestinal tract pathogens. It has reported similarities with ellagic acid, which helps in blood clotting and subsequently reduce bleeding^71^. Further, all these metabolites are reported to be directly absorbed in plasma while administered orally (Supplementary Table S6). After examining the functional significance of the metabolites corresponding to proteins obtained from network analysis, we characterized their biophores to propose repositioning of these metabolites against available drugs.

To determine the set of existing drugs for repositioning in the treatment of pressure-overload LVCH, we performed molecular docking of a set of proteins obtained from network analysis for drug-target protein and metabolite-target protein pairs. The similarity of the biophores for the two pairs of complexes would suggest the interchangeability among available drugs and AR metabolites for therapeutic applications^72,73^. We observed similar biophores for the selected set of 6 target proteins viz. ACADM, COX4I1, HBB, COX6B1, MYH14, and SLC25A4 (Table 2, Supplementary Figs. S2–S7, and Supplementary Table S7). The COX4I1 and COX6B1 corresponded to identical drug and metabolite complexes and individually were found to form similar biophores. However, the protein HBB had two metabolites and corresponding similar drugs. Out of the two, one of the biophores obtained for HBB, namely HBB-cholic acid and HBB-sebacic acid (DrugBank Id: DB07645) complexes were unidentical and hence ignored for further study (Supplementary Fig. S8).

The similar biophores suggest similar biological effects of AR metabolites and their structurally similar drugs (Supplementary Section I and Supplementary Table S8), which will further be investigated for their re-purposing to treat pressure-overload LV cardiac hypertrophy in future studies. Further, we briefly reviewed the biological activity of protein–drug complexes having similar biophores to the protein–AR metabolite complexes, because the information could be useful for the use of the AR metabolites to be potential alternatives to the currently used drugs. The detailed information on the mechanism of these pathways is summarized here.

The first protein–drug complex ACADM-flavin adenine dinucleotide (DrugBank Id: DB03147) complex has a significant role in the Lovastatin pathway and Cerivastatin pathway that are involved in the lowering and inhibition of cholesterol synthesis respectively^74^. The proteins COX4I1 and COX6B1 are part of identical drug and metabolite complexes namely (COX4I1-cholic acid (DrugBank Id: DB02659))—(COX4I1-N-formyl methionine (DrugBank Id: DB04464)), and (COX6B1-cholic acid)—(COX6B1-N-formyl methionine), respectively. The complexes play an important role in cytochrome *c* oxidase activity and regulate bile acid synthesis as well as in the initiation of protein synthesis^75^. The protein–drug complex HBB-dicarboxy naphthalene (DrugBank Id: DB08262) regulates oxygen transporter activity thereby decreasing blood pressure in the body^76^. Next, MYH14-Mant-ADP (DrugBank Id: DB03126) complex helps in Microfilament motor activity that has a major role in myopathy and cytokinesis^77^. The last complex SLC25A4-Adenosine Tri Phosphate (DrugBank Id: DB00171) has a crucial role in metabolism and is also a neurotransmitter. It is involved in adenine transmembrane transporter activity which catalyzes the exchange of cytoplasmic ADP with mitochondrial ATP, across the mitochondrial inner membrane^78^. All these complexes were found to be inhibitors, indicating their role in suppressing one or the other pathways.

Considering the fundamental principles of similar structure similar function^31,32^, we propose that the metabolites namely guanylic acid, cholic acid, L-methionine, and gallic acid corresponding to the protein targets ACADM, COX4I1, COX6B1, HBB, MYH14, and SLC25A4 can be better alternatives to the known drugs as they are extracted from a natural source and have minimal side effects.

## Discussion

The findings of our study indicate the scope for revitalizing Ayurvedic concoctions to be used as therapeutic agents. Using the integrated approach of cheminformatics and network systems biology, we investigated AR, an Ayurvedic rejuvenate for the treatment of cardiovascular diseases (pressure-overload LVCH). The authentic text for the preparation and functional significance of AR is Charaka Chikitsa Sthana, written in the third century BCE. Most of the multi-component formulations in Ayurveda were believed to be acting on multiple targets and therefore, demands a “systems approach”. Such a systematic study could not have been possible without contributory support from in-vivo studies, proteomics analysis, informatics tools, and techniques of network pharmacology. Through this work, we made a conscious attempt to present a complete analytical platform to investigate the efficacy and synergy of ayurvedic medicines systematically.

The in-vivo study on artificially Aorta Constricted and aged Wistar rats provided vital information about changes in protein expression profile, yielding 450 (List L1) and 1166 (List L2) proteins, respectively, that are modulated by AR. The chemical and structural similarity analysis identified structurally similar drugs to AR metabolites, with a turnout of 1004 proteins (List L3). The intersection of protein expression data and structural analysis data (List L4) was used together to construct the PPI network using annotations from existing databases. The network of common proteins was further analyzed for its topological properties such as degree, clustering coefficient, betweenness centrality, etc. The clustering coefficient reveals COX6B1, MYH14, and SLC25A4 proteins that form clique structures or complete subgraphs in the network. The cliques are essential as they form the backbone of a network^50^ and are preserved structures that are responsible for the evolution of any system. They are known as responsible for the robustness^51^ and stability^52^ of the underlying system. Further, the proteins corresponding to nonhub bottlenecks (*H*_*N*_) i.e., nodes having a low degree and high betweenness centrality regime are ACADM, COX4I1, and HBB. The proteins with *H*_*N*_ identify the weak breaking points in the network as they are involved in many pathways. The biological functions of these proteins signify either a regulatory or pathogenic role in the cardiovascular system, such as blood pressure regulation, fatty acid synthesis, cholesterol synthesis, cardiac hypertrophy, cardiomyopathy, and systemic hypertension.

We also constructed and analyzed the metabolite–target–pathway network to determine the synergistic action of AR. The synergy was evaluated qualitatively using the guilt-by-association method^4^ at the metabolite, pathway, and target levels individually to ascertain the combinatorial effect of AR metabolites. Next, the six proteins from network analysis were back-traced to their corresponding AR metabolites and similar drugs. We identified that these existing drug molecules can be repositioned to develop a combinatorial therapy for the treatment of pressure-overload LV cardiac hypertrophy and other cardiovascular diseases.

Though this study details the synergistic action of AR constituents and provides information on the constitution of a repositioned multidrug formulation for the treatment of pressure-overload LVCH, it is limited by the quantitative composition of the repositioned drugs. A possible limitation of this study is the bioavailability of AR metabolites in plasma upon oral administration. However, the plasma absorbance of cholic acid, gallic acid, and L-methionine has been previously reported (Supplementary Table S6). The clinical applications including drug–dose relationship, efficacy considerations, and toxic effects of such drugs need to be rigorously confirmed. A quantitative ratio of the repositioned drugs or even AR constituent metabolites would be required for the further development of combinatorial network medicine. This is the point of exploration for our future studies for developing a therapy for the treatment of pressure-overload LV cardiac hypertrophy, using existing drug molecules and AR constituent metabolites. The analytical approach presented here can be extended further to various other diseases as well. This framework of employing tools of cheminformatics and network biology may provide a direction for developing new drugs, therapeutic targets, biomarkers, and repositioning existing drugs for complex diseases in a time-efficient and cost-effective manner.

## Methods

LC-MS of AR concoction resulted in the identification of 18 metabolites, as significant constituents of AR. The in-vivo experiments provided information containing details of proteins extracted from the proteome analysis (LC-MS) of rat models. The proteome data were recorded for the test (AR treated), placebo or carrier (Ghee-Honey treated), and the control (Untreated) samples in Aorta Constricted and Biologically Aged rats. The 18 metabolites were screened for similar drugs by comparing the structure of AR metabolites with that of drug molecules available in the DrugBank database, employing SMSD and Tc as screening methods. The drugs which are similar to AR metabolites and their associated drug targets were named in List L3. The common proteins among targets from in-vivo study and similarity search (intersection of L1, L2, and L3) resulted in List L4. The PPI network analyses for these common proteins were examined for their network and pathway integrity. Further, we determined the synergy among AR metabolites, their target proteins, and the associated pathways by constructing a multi-component network employing the guilt-by-association method^4^. This network comprised of AR metabolites, their potential protein targets, and associated pathways (MTP network)^4^. The proteins obtained from network analysis were also scrutinized for re-purposing by characterizing their respective biophores, using molecular docking. Detailed procedures employed in this study are the following.

### in-vivo study: data source and acquisition

The in-vivo study of AR on pressure-overload LV cardiac hypertrophy^28^ comprised of the proteomic information of two sets of male Wistar rats (i) AC with pressure-overload LVCH, induced by clipping ascending aorta with titanium clips, and (ii) BA for age-associated cardiac dysfunction. The technique for proteome expression analysis LC-MS was based on the principle that a protein is only detected when it is present above a threshold. The absence of proteins in the control conditions represents the downregulation of those proteins in the disease model. Conversely, the presence of the same proteins in the test condition indicates the upregulation of the proteins due to AR treatment. The objective of our study was to identify proteins, which are directly targeted by the metabolites present in Amalaki Rasayana (AR). Therefore, for our study, we considered all the proteins that are detected from the LC-MS analysis for both the AC and BA rats, resulting in List L1 and L2, respectively. The proteomics analysis had raw files containing details of proteins expressed in all the three sample replicates (AR Treated, Placebo, and Untreated) as RefSeq accessions along with their description, expression scores, etc. For our analysis, we considered only the RefSeq IDs and the expression scores for each sample replicates. Thereafter, we averaged over their expression scores as a representative value for all the replicates (Supplementary Dataset S1). Further, we extracted and converted these RefSeq accessions to protein UNIPROT IDs to deduce the number of proteins for each BA and AC groups as L1 and L2 protein lists (Supplementary Dataset S1).

The in-vivo study was performed at Rajiv Gandhi Center for Biotechnology with the approval of the Institutional animal ethics committee (IAEC) in Rajiv Gandhi Center for Biotechnology (RGCB) under protocol no. IAEC/150/CCK/2012, strictly following the rules and regulations of the Committee for the Purpose of Control and Supervision of Experiments on Animals (CPCSEA), Government of India. The results of this experiment, however, have already been published^28^. In this manuscript, we have only used the proteomics data of the earlier study, and therefore in-vivo studies are not in principle, part of this paper.

### Drug–metabolite similarity search and ontology

The protein targets associated with these drugs (similar to AR metabolites) were considered the representative set of proteins that the AR metabolites can interact with. This is based on the universal philosophy of drug discovery, like structure leads to like functions. To identify potential drug targets for the AR metabolites, we compared the structure of AR metabolites with the structures of well-known modern drug molecules available in the DrugBank database^34^. The similarity index, namely the Tc between drug molecules and metabolites (from AR) were generated using Open Babel^79^. Tc is defined as the ratio of the intersecting set of a molecule to the union set calculated as the measure of similarity^35^. Mathematically, Tc can be represented as Tc (a, b) = *N*_c_/(*N*_a_ + *N*_b_ − *N*_c_), where *N* is the number of attributes in each molecule (a, b) and C is the intersection set.

The pairwise similarity visualization of drug molecules and the AR metabolites was done using SMSD toolkit^36^. Protein ontologies were obtained from KEGG Mapper, Gorilla, and DAVID databases^44–47^.

### Construction and analysis of protein–protein interaction networks

This representative set of proteins play a significant role in therapeutics as they were altered by the treatment with AR as well as targeted corresponding to AR metabolites. Networks can be represented as a collection of nodes connected to their edges. Protein–protein interactions (PPI) from the STRING database were used for the construction of interaction map/network^80^. The *P* value of the interactions was retrieved from the STRING database, which uses a hypergeometric test for statistical significance of the data^80^. Further, the interactions were reconstituted into binary adjacency matrix *(A)* where two proteins, if interacting, were denoted as 1 and 0 if otherwise. Thereafter, topological features, which include structural properties of the network such as degree, clustering coefficient, betweenness centrality, diameter, etc. were calculated. These may reveal the minimal set of drug targets for cardiovascular diseases.

The fundamental topological parameter in a network is the degree of a node (*k*_*i*_), which is defined as the number of first neighbors the node has $(k_i = \mathop {\sum }\limits_j A_{ij})$ ^59^. Another critical parameter is the clustering coefficient (*CC*) of the network. The clustering coefficient of a node *i* (*CC*_*i*_) is defined as the ratio of the number of connections a particular node has and the possible number of connections the particular node can have^49^, as described by Eq. (1).

1 $$CC_i = \frac{{2\mathop {\sum }\nolimits_{j_2 = 1}^{k_i} \mathop {\sum }\nolimits_{j_1 = 1}^{k_i} (A_{ij_1}A_{j_1j_2}A_{j_{2^i}})}}{{k_i(k_i - 1)}}$$

where *i* is the node of interest and *j*_*1*_ and *j*_*2*_ are any two neighbors of the node *i* and *k*_*i*_ are the degrees of the node *i*. The average clustering coefficient (*<CC*>) of a network is described by Eq. (2)

2 $$CC = \frac{1}{n}\mathop {\sum }\limits_{i = 1}^n CC_i$$

These are also known as cliques. They are complete subgraphs in the network which are known to be the conserved part of the network^50^. The average clustering coefficient of the network characterizes the overall tendency of nodes to form clusters or groups. Further, the diameter of the network measures the longest of the shortest path between all the pairs of nodes^40^. It gives information on how fast a signal can transmit to the whole network.

Next, the betweenness centrality *(β*_*c*_*)* of a node *i* is defined as the fraction of the shortest paths between node pairs that pass through the said node of interest^59^, as described by Eq. (3)

3 $$\beta _{ci} = \mathop {\sum}\limits_{st} {\frac{{n_{st}^i}}{{g_{st}}}}$$

where, *n*^*i*^_*st*_ is the number of paths from *s* to *t* that passes through *i* and *g*_*st*_ is the total number of paths from *s* to *t* in the network.

### Synergistic effects of AR constituents

The use of drug combinations against multiple linked targets in a disease-specific network is a more sensible approach for the treatment of complex diseases. Ayurveda is the science of combinatorial therapy and its medicines often have multiple constituents, which are considered to operate through a cooperative mechanism termed synergy. Most of the research on combinatorial medicines has been based on the hypothesis that the synergistic action of drugs is due to their combinatorial effects on targets identified by their network topological features. Thus, we categorized the proteins in accordance with their corresponding metabolites and observed the presence of proteins and their corresponding metabolites and pathways in the maintenance of network integration. We use the guilt-by-association approach^4^ where the distance of ≤3 among the connected entities was taken into consideration for each dataset.

### Identification of binding sites: molecular docking for re-purposing

The proteins obtained from network analysis were back-traced to their metabolites and drug targets for identification of the binding sites among the protein–metabolite and protein–drugs through molecular docking using AutoDock Vina^81^. To perform this exercise, the structures of the proteins were identified from Protein Data Bank (PDB)^82^. Thereafter, the selected proteins retrieved from PDB were using Chimera separated from the ligands and chains that are not relevant to the present study. The water molecules were also removed by using AutoDockTools (ADT)^81^. This was followed by the addition of Gasteiger charges and adjusting the charges on each residue to bring integral charges across the macromolecule. Hydrogen atoms were added, the non-polar ones were merged, and the macromolecule thus obtained was saved as a PDBQT file. Next, the ligand structures were retrieved from DrugBank and PubChem databases^34^. They were energy minimized using Avogadro’s 1.2.0 steepest descent algorithm to reduce any bad conformations that may be introduced and give artefacts of potential energy. It is likely that these conformations were not preserved when docking was finally carried out since the ligand is treated as a flexible entity in AutoDock Vina. It was read into ADT, torsion tree root detected, and processed as another PDBQT file. This modified structure (preparatory files) was then further docked blindly.

The strategy behind using blind docks relied on the fact that we can scale the exhaustiveness with grid box and allow the docking to be completed faster, along with generally better results utilizing the system’s multithreaded architecture^81^. The docking was run and examined using LigPlot and PyMol for screening the biophores of the docked protein and ligand (drug/metabolite)^83^. Further, we generated a biophore fingerprint table to identify similar (recurring) residues in the binding pocket of protein–metabolite and protein–drug complexes.

### Reporting summary

Further information on experimental design is available in the Nature Research Reporting Summary linked to this paper.

## Supplementary information

Supplementary Information

Dataset S1

nr reporting summary

## Data Availability

All the relevant data supporting the findings of this study are included in the paper and its Supplementary Information files. Processed data in the form of figures and tables are presented in the main paper. The Supplementary Information contains two files: (1) Supplementary Information file with biophore details, network topologies, and information related to identified targets. (2) Supplementary Dataset S1: This file has details of target profiling of AC and BA rat models, Tanimoto coefficient (*T*c) of drugs with respect to their similar metabolites, common protein expression data, etc.

## Code availability

For this study, no custom made codes and algorithms were used.
