# Supplementary material for: Mapping drug-target interactions and synergy in multi-molecular therapeutics for pressure-overload cardiac hypertrophy
Source: NPJ Syst Biol Appl. 2021 Feb 15;7:11. doi: 10.1038/s41540-021-00171-z (PMC7884732; doi:10.1038/s41540-021-00171-z)
Supplement: Supplementary file 1 — Supplementary Information [file 41540_2021_171_MOESM1_ESM.pdf]

Mapping Drug-Target Interaction and Synergy in Multi-molecular Therapeutics for Pressure Overload Cardiac Hypertrophy

Aparna Rai[1], Vikas Kumar[2], Gaurav Jerath[1,3], CC Kartha[4,*] and Vibin Ramakrishnan[1,*]

[1] Molecular Informatics and Design Laboratory, Department of Biosciences and Bioengineering, Indian Institute of Technology Guwahati, Guwahati, Assam 781039, India.

[2] Laboratory of Cardiovascular Science, National Institute on Aging, National Institutes of Health, Baltimore, MD 21224, USA.

[3] PepThera Laboratories Private Limited, Guwahati, Assam 781014, India.

[4] Society for Continuing Medical Education & Research, Kerala Institute of Medical Sciences, Thiruvananthapuram 695029, India.

* To whom correspondence should be addressed

Email: cckartha@gmail.com, vibin@iitg.ac.in

<u>Supplementary Information</u>

The supplementary information file includes:

Supplementary Information text
Supplementary Figure. S1. Gallic and Ellagic acid in AR
Supplementary Figure S2. Biophore Fingerprints for ACADM
Supplementary Figure. S3. Biophore Fingerprints for COX4I1
Supplementary Figure. S4. Biophore Fingerprints for COX6B1
Supplementary Figure. S5. Biophore Fingerprints for HBB with Gallic acid
Supplementary Figure. S6. Biophore Fingerprints for MYH14
Supplementary Figure. S7. Biophore Fingerprints for SLC25A4
Supplementary Figure. S8. Biophore Fingerprints for HBB with Cholic acid
Supplementary Table S1. AR Metabolites
Supplementary Table S2. Peak Intensities
Supplementary Table S3. Topological Characteristics of PPI network
Supplementary Table S4. Pathway Analysis for L4 proteins
Supplementary Table S5. Similarity Search Data
Supplementary Table S6. Plasma Absorbance of AR metabolites
Supplementary Table S7. Binding Complexes of Protein-Drug/Metabolite
Supplementary Table S8. Biophore Fingerprint Matrix
Legend for extra file (Supplementary Dataset S1)
Supplementary References

Other supplementary materials for this manuscript include the following:

Supplementary Dataset S1. *In-vivo* data, Similarity search, Proteins lists and PPI interactions

Supplementary Information Text

I. Drug Repositioning for Cardiac Hypertrophy

- **Molecular Docking: Binding complexes**

The similar structure-function theory described in the above sections relies on the binding of ligands (drugs and metabolites) to their respective receptors (proteins/drug-targets). For a drug to exert similar or identical action of its structurally similar metabolite or drug, should bind to the same site on the receptor protein/ drug target [1]. The combination of the drug pharmacophore and the target binding site residues is referred to as a biophore [2]. Therefore, we used molecular docking to identify the similar biophores for determining the drug molecules, which can be repositioned for use in cardiovascular diseases. The set of important target proteins from the topological analysis of the protein-protein interaction network were used in this study.

We performed blind docking for the metabolites and their structurally similar drugs by incorporating the entire protein in the docking grid, using AutoDock Vina [3]. The docked complexes were analyzed for similarity (recurring residues) of their respective biophores and further, investigated for known interactions and biological activity of the protein targets used for molecular docking. Such information is crucial for ascertaining the utility of metabolites of AR as potential alternates to currently used therapeutic drugs targeted against identical protein targets. Conversely, the drugs similar to metabolites can also be repositioned for their combinatorial use in the treatment of cardiac hypertrophy. The list of metabolites, their similar drugs and associated target proteins used for molecular docking are given in Table 1 of the main manuscript. The biophores obtained for each of the protein-drug and their corresponding protein-metabolite complexes had similar binding pockets except for HBB-Cholic acid and HBB-Sebacic acid complexes.

The drug-receptor and metabolite-receptor complexes obtained through a blind docking protocol are given in Figures. S2-S8. The complete biophore fingerprint of recurring and non-recurring residues for each of the protein complexes are enlisted in (Table S8).

<u>**Supplementary Figures**</u>

1. Gallic and Ellagic acid in AR:

a)

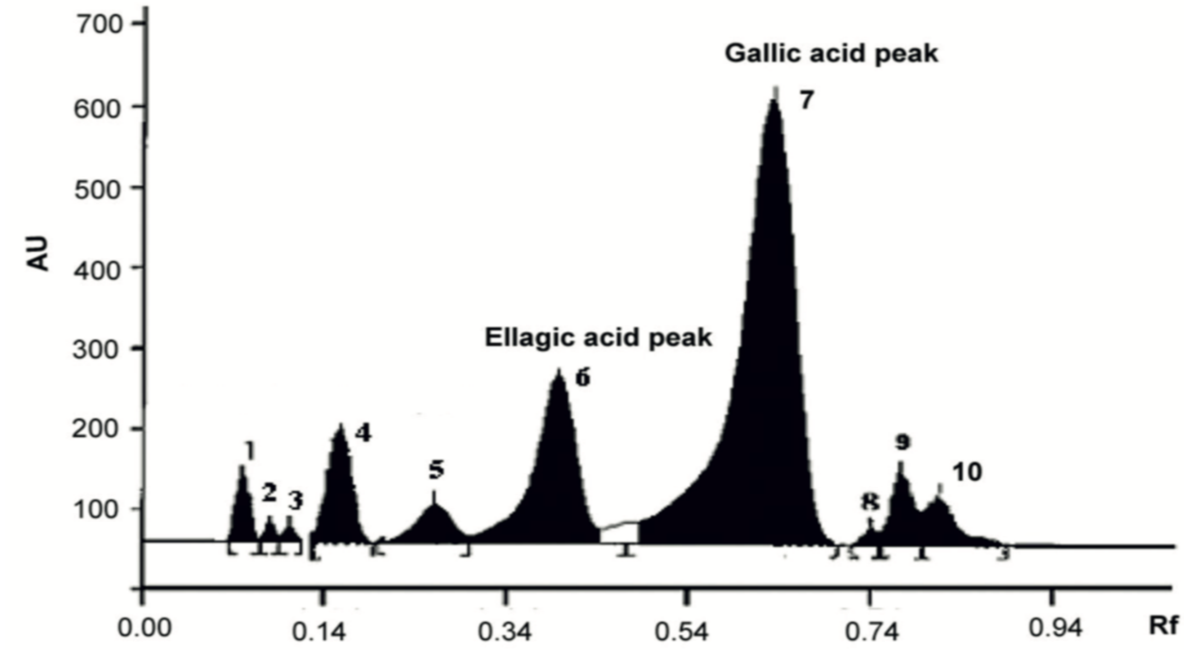

b)

S. No.	Metabolite	(%w/w)
1.	Gallic Acid	1.5
2.	Ellagic Acid	0.40

Supplementary Figure S1: Gallic and Ellagic acid in AR. a) The HPTLC chromatogram shows the peaks identified as Gallic acid and Ellagic acid found in the AR concoction. b) The table shows the concentration of Gallic and Ellagic acid in AR as a weight by weight percentage.

2. ACADM-Guanylic Acid-FAD:

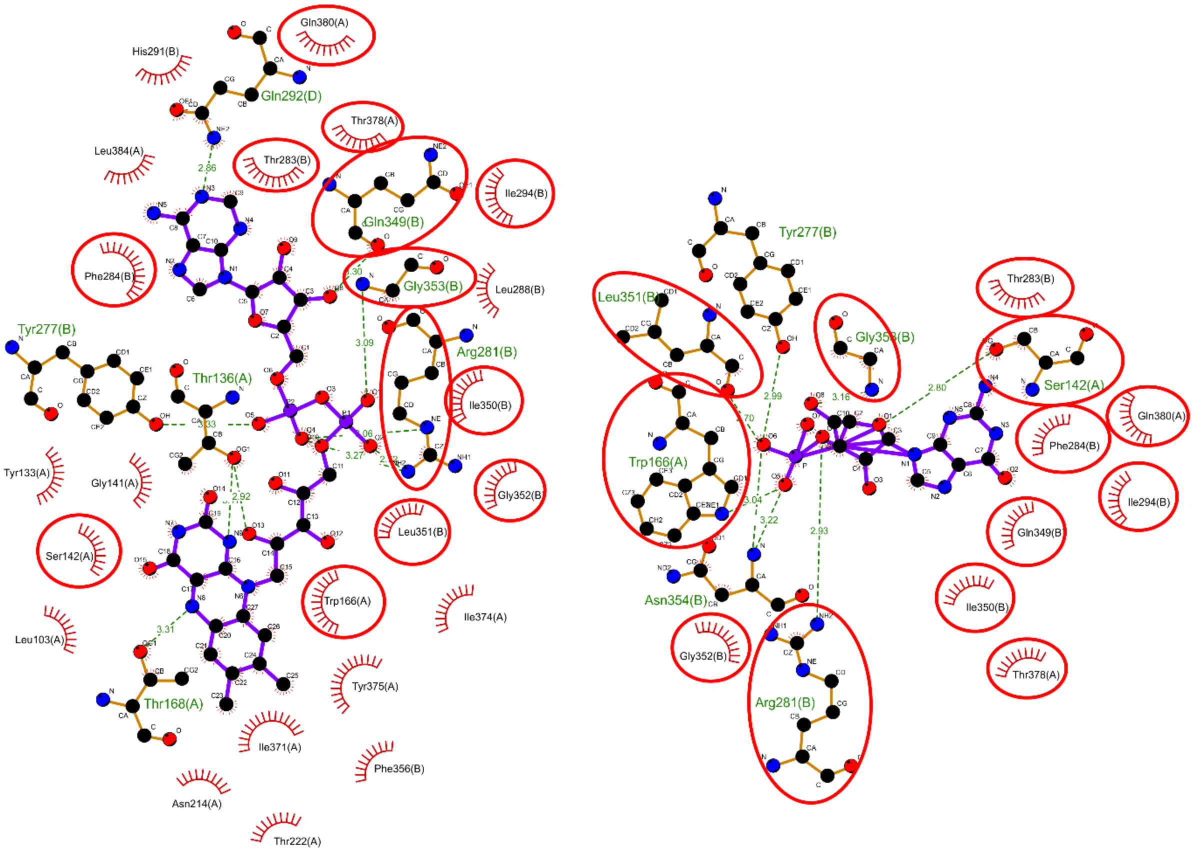

ACADM with FLAVIN ADENINE DINUCLEOTIDE ACADM with GUANYLIC ACID

Supplementary Figure S2: Biophore Fingerprints for ACADM: ACADM protein was docked with Flavin Adenine Dinucleotide (FAD) and guanylic acid (AR metabolite). The docking of the two ligands with ACADM resulted in identical biophoric signatures. The ligands interact with 13 identical amino acid residues of ACADM. The common residues have been encircled in the two complexes for comprehension. This suggests the common biological effects of the two ligands (FAD and guanylic acid).

3. COX4I1-L-methionine-L-formyl Methionine:

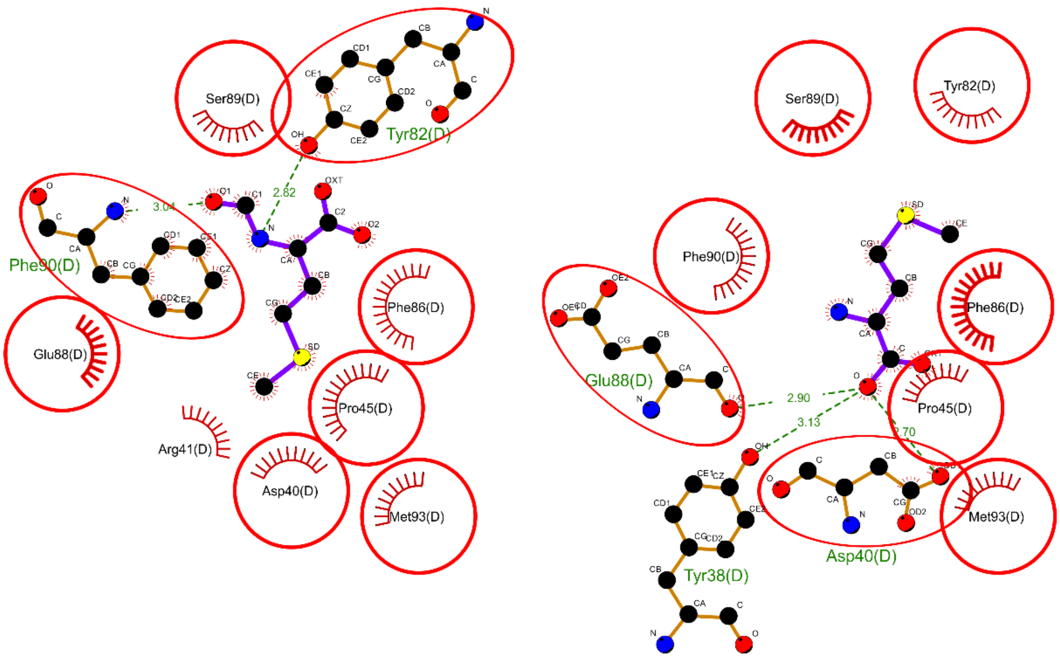

COX4I1 with N-FORMYL METHIONINE COX4I1 with L-METHIONINE

Supplementary Figure S3: Biophore Fingerprints for COX4I1: COX4I1 protein was docked with N-Formyl Methionine and L-methionine (AR metabolite). The docking of the two ligands with COX4I1 resulted in identical biophoric signatures. The ligands interact with 8 identical amino acid residues of COX4I1. The common residues have been encircled in the two complexes for comprehension. This suggests the common biological effects of the two ligands (N-Formyl Methionine and L-methionine).

4. COX6B1- L-methionine-L-formyl Methionine:

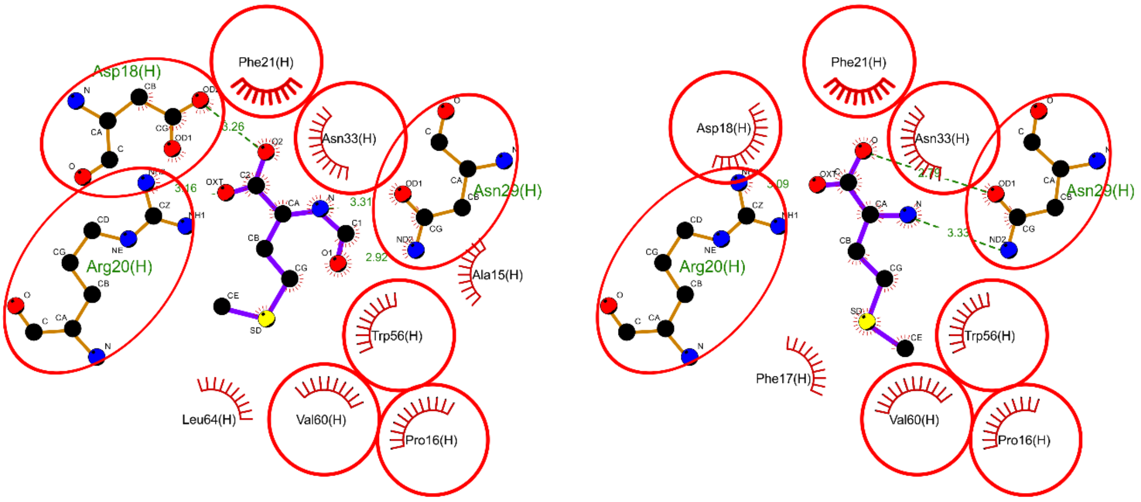

COX6B1 with N-FORMYL METHIONINE   COX6B1 with L-METHIONINE

Supplementary Figure S4: Biophore Fingerprints for COX6B1: COX6B1 protein was docked with N-Formyl Methionine and L-methionine (AR metabolite). The docking of the two ligands with COX6B1resulted in identical biophoric signatures. The ligands interact with 8 identical amino acid residues of COX6B1. The common residues have been encircled in the two complexes for comprehension. This suggests the common biological effects of the two ligands (N-Formyl Methionine and L-methionine).

5. HBB-Gallic Acid-2,6-Dicarboxy Napthalene:

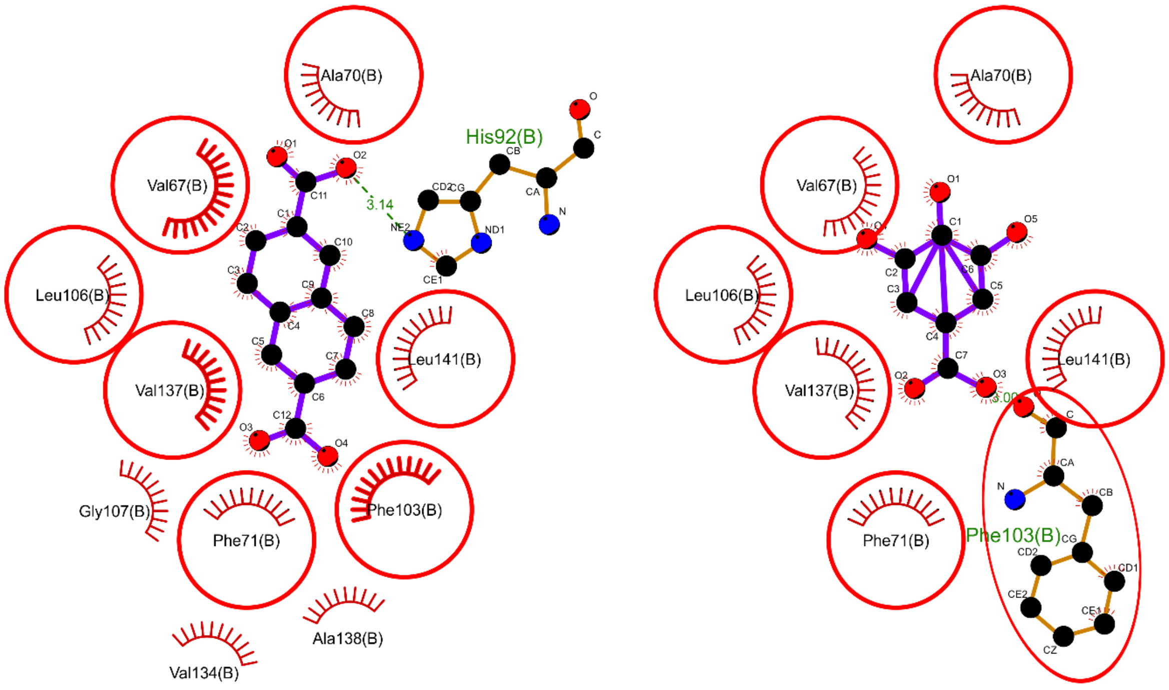

HBB with 2,6-DICARBOXY NAPTHALENE HBB with GALLIC ACID

Supplementary Figure S5: Biophore Fingerprints for HBB with Gallic Acid: HBB protein was docked with 2,6-Dicarboxy naphthalene and gallic acid (AR metabolite). The docking of the two ligands with HBB resulted in identical biophoric signatures. The ligands interact with 7 identical amino acid residues of HBB. The common residues have been encircled in the two complexes for comprehension. This suggests the common biological effects of the two ligands.

6. MYH14-Guanylic Acid-ADP:

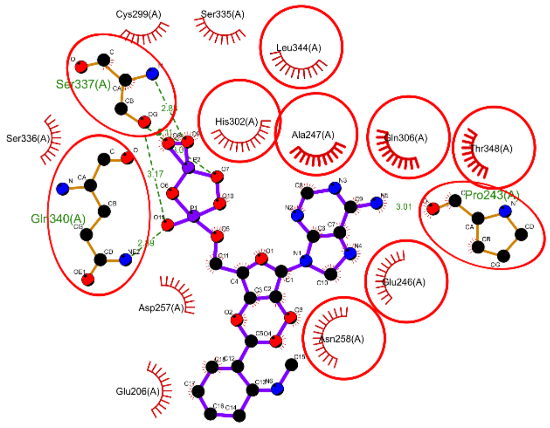

MYH14 with MANT-ADENOSINE DI PHOSPHATE

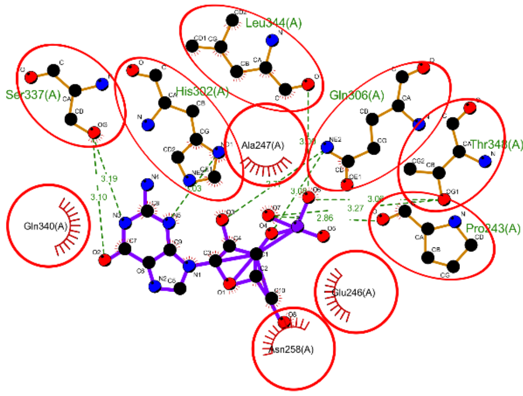

MYH14 with GUANYLIC ACID

Supplementary Figure S6: Biophore Fingerprints for MYH14: MYH14 protein was docked with Mant-Adenosine di-phosphate and guanylic acid (AR metabolite). The docking of the two ligands with MYH14 resulted in identical biophoric signatures. The ligands interact with 10 identical amino acid residues of MYH14. The common residues have been encircled in the two complexes for comprehension. This suggests the common biological effects of the two ligands.

7. SLC25A4-Guanylic Acid-ATP:

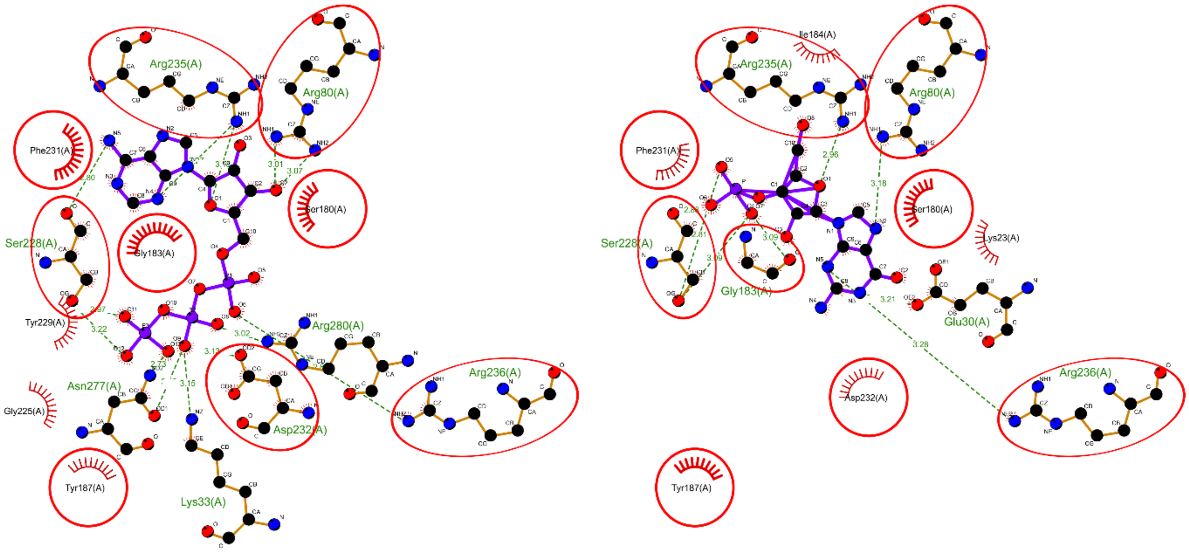

SLC25A4 with ADENOSINE TRI PHOSPHATE SLC25A4 with GUANYLIC ACID

Supplementary Figure S7: Biophore Fingerprints for SLC25A4: SLC25A4 protein was docked with Adenosine tri-phosphate and guanylic acid (AR metabolite). The docking of the two ligands with SLC25A4 resulted in identical biophoric signatures. The ligands interact with 9 identical amino acid residues of SLC25A4. The common residues have been encircled in the two complexes for comprehension. This suggests the common biological effects of the two ligands.

8. HBB-Cholic Acid-Sebacic Acid:

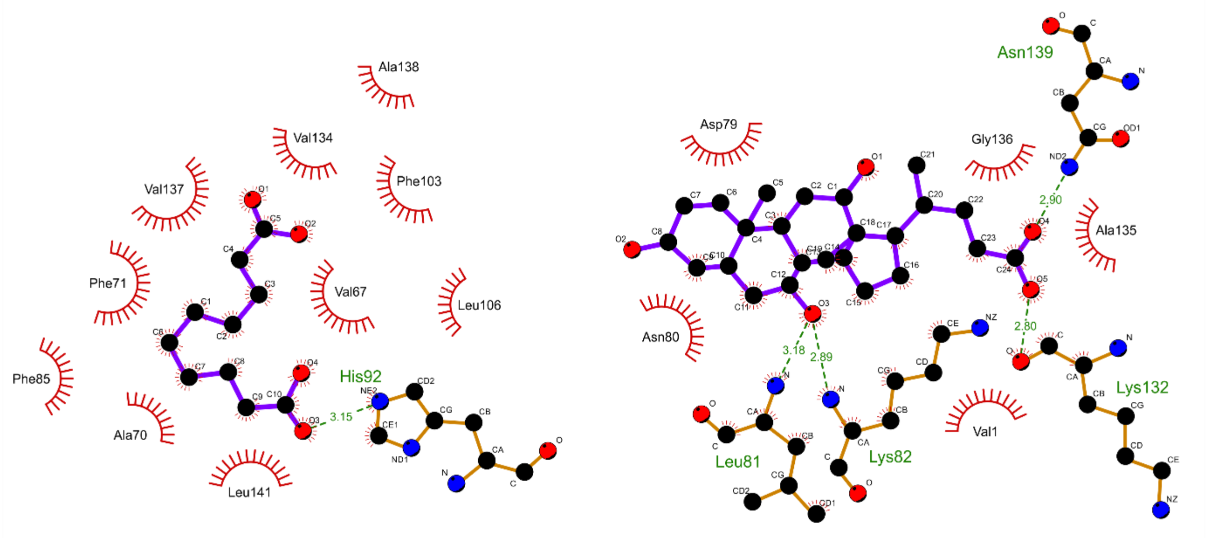

HBB with Sebacic Acid HBB with Cholic Acid

Supplementary Figure S8: Biophore Fingerprints for HBB with Cholic Acid: HBB protein was docked with Sebacic acid and Cholic acid (AR metabolites). The docking of the two ligands with HBB resulted in non-identical biophores. No identical amino acids were identified in the two biophores, suggesting different binding sites and action for the two metabolites.

Supplementary Tables

1. **Supplementary Table S1: AR Metabolites (other than primary constituent's Gallic acid and Ellagic acid).** The table lists metabolites (in addition to Gallic acid and Ellagic acid) that are present in the AR concoction. These metabolites were obtained from the LC-MS analysis.

S. No.	Metabolite	m/z value	Identifier Peak
1.	Biocytin	373.192	M + H[1+]
2.	Methylcobalamin	1382.5472	M + K[1+]
3.	L-Methionine	150.0575	M + H[1+]
4.	Pyridoxamine Phosphate	249.0617	M + H[1+]
5.	Prostaglandin B1	359.2163	M + Na[1+]
6.	4-Hydroxy-all-trans-retinyl_acetate	328.218	M-NH 3 + H[1+]
7.	13'-carboxy-alpha-tocotrienol	454.3103	M + H[1+]
8.	Cholic Acid	409.2919	M + H[1+]
9.	1alpha,24R,25-trihydroxyvitamin D3 (Calcitetrol)	455.3133	M + Na[1+]
10.	Guanylic Acid	402.0196	M + K[1+]
11.	Carbamoyl Phosphate	141.9899	M + H[1+]
12.	5-formiminotetrahydrofolate	473.1939	M + H[1+]
13.	Nicotinate D-ribonucleoside	279.0692	M + Na[1+]
14.	Sulfate derivative of norepinephrine (Noradrenaline Sulfate)	250.0401	M + H[1+]
15.	12-oxo-20-dihydroxy-leukotriene_B4	388.1884	M + Na[1+]
16.	5S,6S-epoxy-15S-hydroxy-7E,9E,11Z,13E-eicosatetraenoic acid	334.2144	M + H[1+]

2. **Supplementary Table S2: Peak Intensities.** The table details the peak intensities of other metabolites present in AR concoction and retention time.

S. No.	Metabolite	Retention Time (mins)	Maximum Peak intensity
1.	Biocytin	14.3	5.38E + 03
2.	Methylcobalamin	0.68	3.93E + 03
3.	L-Methionine	15.54	2.93E + 03
4.	Pyridoxamine Phosphate	8.72	1.22E + 03
5.	Prostaglandin B1	15.44	1.83E + 03
6.	4-Hydroxy-all-trans-retinyl_acetate	6.31	6.74E + 02
7.	13'-carboxy-alpha-tocotrienol	15.63	1.54E + 04
8.	Cholic Acid	15.51	4.96E + 03
9.	1alpha,24R,25-trihydroxyvitamin D3 (Calcitetrol)	15.63	4.43E + 03
10.	Guanylic Acid	7.98	1.04E + 03
11.	Carbamoyl Phosphate	18.29	2.09E + 03
12.	5-formiminotetrahydrofolate	13.61	9.78E + 02
13.	Nicotinate D-ribonucleoside	0.79	1.71E + 03
14.	Sulfate derivative of norepinephrine (Noradrenaline Sulfate)	14.72	7.82E + 02
15.	12-oxo-20-dihydroxy-leukotriene_B4	14.33	1.09E + 04
16.	5S,6S-epoxy-15S-hydroxy-7E,9E,11Z,13E-eicosatetraenoic acid	13.64	2.40E + 03

3. **Supplementary Table S3: Topological Characteristics of PPI network.** The PPI network for list of proteins (L4) identified from the intersection of lists L1, L2 and L3, was analyzed for different topological properties such as betweenness centrality, clustering coefficient and degree given below.

Protein/ Target	Degree	Clustering Coefficient	Betweenness Centrality
PRKAR1A	0	0	0
NNT	0	0	0
HBB	2	0	0.05555556
HBA1	1	0	0
PGK1	17	0.32352941	0.10014426
GAPDH	21	0.24285714	0.34199597
DLAT	6	0.66666667	0.03527007
OGDH	7	0.80952381	0.00333168
COX4I1	6	0.33333333	0.11557687
COX6B1	2	1	0
MDH1	14	0.37362637	0.05803491
GOT1	7	0.52380952	0.00922991
PYGB	6	0.86666667	0.00111413
PYGM	7	0.76190476	0.00322977
GOT2	10	0.42222222	0.0504836
PYGL	6	0.86666667	0.00111413
LDHA	15	0.39047619	0.09450452
SOD1	12	0.36363636	0.07175438
EEF2	7	0.42857143	0.06280045
EIF2S3	1	0	0
ACSS1	2	0	0.00171958
ACADM	7	0.14285714	0.08419521
NME2	9	0.33333333	0.02873425
AK2	5	0.5	0.00531935
HINT1	5	0.6	0.00272404
FKBP1A	4	0.5	0.00655695
HPRT1	5	0.8	0.00305922
IVD	2	0	0.004355
ACTA1	7	0.38095238	0.05667962
CALM1	10	0.26666667	0.07244107
ACADS	1	0	0
AK1	4	0.5	0.00101023
YWHAE	5	0.7	0.00265419
DBI	4	0.16666667	0.01619864
GC	2	0	0.00147977
CRP	4	0.33333333	0.02350986
AKR1B1	5	0.7	0.00427041
MYH14	2	1	0
SLC25A4	2	1	0
CA	1	0.1	0

4. **Supplementary Table S4: Pathway Analysis for L4 proteins.** The pathways associated with L4 list of proteins shown as nodes in the PPI network (Figure. 2 of manuscript). The ontology done using STRING database having false discovery rate (FDR) <0.05.

Pathway	Proteins involved	FDR
Metabolic pathways	ACADM, ACADS, ACSS1, AK1, AK2, AKR1B1, COX4I1, COX6B1, DLAT, GAPDH, GOT1, GOT2, HPRT1, IVD, LDHA, MDH1, NME2, NNT, OGDH, PGK1, PYGB, PYGL, PYGM	1.28E-15
Carbon metabolism	ACADM, ACADS, ACSS1, DLAT, GAPDH, GOT1, GOT2, MDH1, OGDH, PGK1	3.16E-12
Glycolysis / Gluconeogenesis	ACSS1, DLAT, GAPDH, LDHA, PGK1	1.30E-05
Pyruvate metabolism	ACSS1, DLAT, LDHA, MDH1	4.54E-05
Glucagon signaling pathway	CALM1, LDHA, PYGB, PYGL, PYGM	4.76E-05
Cysteine and methionine metabolism	GOT1, GOT2, LDHA, MDH1	4.76E-05
Insulin signaling pathway	CALM1, PRKAR1A, PYGB, PYGL, PYGM	1E-04
Biosynthesis of amino acids	GAPDH, GOT1, GOT2, PGK1	2E-04
Citrate cycle (TCA cycle)	DLAT, MDH1, OGDH	5E-04
Starch and sucrose metabolism	PYGB, PYGL, PYGM	5E-04
Propanoate metabolism	ACADM, ACSS1, LDHA	5E-04
Phenylalanine, tyrosine and tryptophan biosynthesis	GOT1, GOT2	7E-04
Valine, leucine and isoleucine degradation	ACADM, ACADS, IVD	0.0012
Necroptosis	PYGB, PYGL, PYGM, SLC25A4	0.0023
Alzheimer's disease	CALM1, COX4I1, COX6B1, GAPDH	0.0029
Purine metabolism	AK1, AK2, HPRT1, NME2	0.003
Thiamine metabolism	AK1, AK2	0.003
Phenylalanine metabolism	GOT1, GOT2	0.004
2-Oxocarboxylic acid metabolism	GOT1, GOT2	0.004
Huntington's disease	COX4I1, COX6B1, SLC25A4, SOD1	0.004
Arginine biosynthesis	GOT1, GOT2	0.0046
HIF-1 signaling pathway	GAPDH, LDHA, PGK1	0.0054
Insulin resistance	PYGB, PYGL, PYGM	0.0064
African trypanosomiasis	HBA1, HBB	0.0103
Alanine, aspartate and glutamate metabolism	GOT1, GOT2	0.0104
Tyrosine metabolism	GOT1, GOT2	0.0106
Parkinson's disease	COX4I1, COX6B1, SLC25A4	0.012
Fatty acid degradation	ACADM, ACADS	0.0144
Malaria	HBA1, HBB	0.0157
Arginine and proline metabolism	GOT1, GOT2	0.0158
Fatty acid metabolism	ACADM, ACADS	0.0158
PPAR signaling pathway	ACADM, DBI	0.0318
Drug metabolism - other enzymes	HPRT1, NME2	0.0332
Cardiac muscle contraction	COX4I1, COX6B1	0.0332

5. **Supplementary Table S5: Similarity Search Data.** Associated Drug Targets (Proteins), AR constituents and Structurally Similar Drugs with their similarity scores.

S.No.	PROTEIN	METABOLITE	SIMILAR DRUGS	TANIMOTO COEFFICIENT (Tc)
1.	ACADM	Guanylic Acid	DB03147	0.72
2.	ACADS	Guanylic Acid	DB03147	0.72
3.	ACSS1	Guanylic Acid	DB00131, DB00171	0.79, 0.72
4.	ACTA1	Guanylic Acid	DB04395	0.66
5.	AK1	Guanylic Acid	DB01717	0.717
6.	AK2	Guanylic Acid	DB01717	0.717
7.	AKR1B1	Guanylic Acid	DB03461	0.66
8.	CA2 (CAR2)	Ellagic acid	DB08846	1
9.	CALM1	Cholic Acid	DB08231	0.86
10.	COX4I1	Cholic Acid, L-Methionine	DB02659, DB04464	1, 0.697
11.	COX6B1	Cholic Acid, L-Methionine	DB02659, DB04464	1, 0.697
12.	CRP	Guanylic Acid	DB03945	0.6, 0.6
13.	DBI	Guanylic Acid	DB01992	0.64
14.	DLAT	Cholic Acid	DB03760	0.63
15.	EEF2	Guanylic Acid	DB04315, DB02059	0.94, 0.73
16.	EIF2S3	Guanylic Acid	DB04315	0.94
17.	FKBP1A	Cholic Acid	DB08231	0.86
18.	GAPDH	Guanylic Acid	DB02059, DB03893	0.73, 0.65
19.	GC	Calcitetrol	DB00136	0.98
20.	GOT1	Pyridoxamine Phosphate	DB00114	0.84
21.	GOT2	Pyridoxamine Phosphate	DB00114, DB02783	0.84, 0.79
22.	HBA1	Gallic Acid, Cholic Acid	DB08262, DB07645	0.61, 0.86
23.	HBB	Gallic Acid, Cholic Acid	DB08262, DB07645	0.61, 0.86
24.	HINT1	Guanylic Acid	DB01972, DB00131	1, 0.79
25.	HPRT1	Guanylic Acid	DB02309	0.68
26.	IVD	Guanylic Acid	DB04036	0.6
27.	LDHA	Guanylic Acid	DB02483	0.67
28.	MDH1	Guanylic Acid	DB03461	0.66
29.	MYH14	Guanylic Acid	DB03126	0.63
30.	NME2	Guanylic Acid	DB04315	0.94
31.	NNT	Guanylic Acid	DB03461, DB01763	0.66, 0.63
32.	OGDH	Cholic Acid	DB00313	0.77
33.	PGK1	Guanylic Acid	DB03909	0.67
34.	PRKAR1A	Guanylic Acid	DB02315, DB02527	0.94, 0.75
35.	PYGB	Pyridoxamine Phosphate	DB00114	0.84
36.	PYGL	Guanylic Acid, Pyridoxamine Phosphate	DB00131, DB00114	0.79, 0.84
37.	PYGM	Guanylic Acid,, Pyridoxamine Phosphate	DB04566, , DB00114, DB04083	0.91,, 0.84, 0.64
38.	SLC25A4	Guanylic Acid	DB00171	0.72
39.	SOD1	Noradrenaline Sulfate	DB00668, DB01064	0.62, 0.60
40.	YWHAE	Carbamoyl Phosphate	DB01780	0.66

6. **Supplementary Table S6: Plasma Absorbance of AR metabolites.** The table reports previous studies available on the candidate metabolites on absorbance in plasma upon oral administration. These candidate metabolites correspond to the proteins resultant from the network analysis of L4 proteins and have potential therapeutic importance in the treatment of Left Ventricular Cardiac Hypertrophy (LVCH).

S. No.	Candidate metabolite	Absorbance in Plasma	Reference
1.	Guanylic Acid	As per the ADMET predictions of the DrugBank database, guanylic acid has the probability of 71% of absorbance from intestines and a 95% probability of crossing the blood brain barrier.	[4]
2.	Cholic Acid	Cholic Acid is an approved drug and orally administered for the treatment of bile acid synthesis disorders and peroxisomal disorders. This suggests it is absorbed in the plasma.	[5]
3.	L-Methionine	The supplementation of L-methionine increases the plasma absorbance of other metabolites.	[6]
4.	Gallic Acid	Pharmacokinetic analysis revealed 4-O-methylgallic acid (4OMGA) as the major metabolite in human plasma.	[7]

7. Supplementary Table S7: Binding Complexes of Protein-Drug/Metabolite. The binding complexes studied using molecular docking for analyzing the biophore associated for each set of AR metabolite and similar drug molecule with a common target protein are given. The PDB ID for the target protein is also provided. All AR metabolites have been categorized as Experimental molecules while their similar drugs have been categorized as approved/investigational as per their clinical trial outcome/process.

S No.	Complexes	Drug name	PDB ID	Category
1	ACADM-DB03147	FAD (Flavin Adenine Dinucleotide)	2A1T	Approved
2	ACADM-GUAN	Guanylic acid	2A1T	Experimental
3	COX4I1-CHOLIC	Cholic Acid	5Z62	Approved
4	COX4I1-DB04464_L	N-formyl methionine	5Z62	Experimental
5	COX4I1-LMETH	L-methionine	5Z62	Approved, Nutraceutical
6	COX6B1-CHOLIC	Cholic Acid	5Z62	Approved
7	COX6B1-DB04464_L	N-formyl methionine	5Z62	Experimental
8	COX6B1-LMETH	L-methionine	5Z62	Approved, Nutraceutical
9	HBB-CHOLIC	Cholic Acid	2DN2	Approved
10	HBB-DB07645_C	Sebacic Acid	2DN2	Experimental
11	HBB-DB08262_GA	2,6-dicarboxy naphthalene	2DN2	Experimental
12	HBB-GALLIC	Gallic acid	2DN2	Approved
13	MYH14-DB03126	Mant-Adp	5I4E	Experimental
14	MYH14-GUAN	Guanylic acid	5I4E	Experimental
15	SLC25A4-DB00171	ATP (Adenosine triphosphate)	1OKC	Investigational, Nutraceutical
16	SLC25A4-GUAN	Guanylic acid	1OKC	Experimental

8. **Supplementary Table S8: Biophore Fingerprint Matrix.** The Table showing similarity among the binding pockets of protein-drug and protein-metabolite complexes. Each of the column represents the residues that comprise the biophore followed by rows representing the complexes. The table is shown in a binary format, *0* indicating the non-recurring residues and *1* indicating the recurring ones.

ACADM Residues	Arg 281	Asn 354	Asn 214	Gln 292	Gln 349	Gln 380	Gly 141	Gly 352	Gly 353	His 291	Ile 294	Ile 350	Ile 371	Ile 374	Leu 103	Leu 288	Leu 351	Leu 384	Phe 284	Phe 356	Ser 142	Thr 136	Thr 168	Thr 222	Thr 283	Thr 378	Trp 166
Metabolite	1	1	0	0	1	1	0	1	1	0	1	1	0	0	0	0	1	0	1	0	1	0	0	0	1	1	1
Similar Drug	1	0	1	1	1	1	1	1	1	1	1	1	1	1	1	1	1	1	1	1	1	1	1	1	1	1	1
COX4I1 Residues	Ser 89	Tyr 82	Phe 86	Pro 45	Met 93	Asp 40	Arg 41	Glu 88	Phe 90	Tyr 38																	
Metabolite	1	1	1	1	1	1	0	1	1	1																	
Similar Drug	1	1	1	1	1	1	1	1	1	0																	
COX6B1 Residues	Asp 18	Phe 21	Asn 33	Asn 29	Ala 15	Trp 56	Pro 16	Val 60	Leu 64	Arg 20	Phe 17																
Metabolite	1	1	1	1	0	1	1	1	0	1	1																
Similar Drug	1	1	1	1	1	1	1	1	1	1	0																
HBB Residues	Ala 70	His 92	Leu 141	Phe 103	Ala 138	Val 134	Phe 71	Gly 107	Val 137	Leu 106	Val 67																
Metabolite	1	0	1	1	0	0	1	0	1	1	1																
Similar Drug	1	1	1	1	1	1	1	1	1	1	1																
MYH14 Residues	Ser 377	His 302	Leu 344	Ala 247	Gln 306	Thr 348	Pro 243	Glu 246	Asn 258	Gln 340	Cys 299	Ser 335	Asp 257	Glu 206	Ser 336												
Metabolite	0	0	1	1	1	1	1	1	1	1	0	0	0	0	0												
Similar Drug	1	1	1	1	1	1	1	1	1	1	1	1	1	1	1												
SLC25A4 Residues	Arg 235	Arg 80	Ser 180	Arg 280	Arg 236	Asp 232	Lys 33	Tyr 187	Gly 225	Asn 277	Tyr 229	Ser 228	Phe 231	Gly 183	Ile 184	Lys 23	Glu 30										
Metabolite	1	1	1	0	1	1	0	1	0	0	0	1	1	1	1	1	1										
Similar Drug	1	1	1	1	1	1	1	1	1	1	1	1	1	1	0	0	0										

<u>Legend for extra files (Dataset S1)</u>

1. **Supplementary Dataset S1: *In-vivo* data, Similarity search, Proteins lists and PPI interactions.** The dataset tabulates the lists of proteins with their expression profiles from *in-vivo* experiments for Aorta Constricted and Biologically Aged rat models. Additionally, the dataset S1 enlists L1 (AC) and L2 (BA), targets associated with metabolites and their similar drugs (L3) and the intersection of L1, L2 and L3 proteins as common proteins (L4). It also gives information on the similar drugs associated with all the 18 AR metabolites and targets corresponding to them. The metabolites associated with common proteins (L4), their similar drugs and other related details such as Tc score, the status of drugs (approved, experimental, investigational) etc. are also found. The dataset S1 also shows of the network connections of the L4 proteins which were further analyzed for various network topologies.

<u>**Supplementary References**</u>

1 Berger, S. I. & Iyengar, R. Role of systems pharmacology in understanding drug adverse events. *Wiley Interdiscip Rev Syst Biol Med* **3**, 129-135, doi:10.1002/wsbm.114 (2011).

2 Mehra, A., Jerath, G., Ramakrishnan, V. & Trivedi, V. Characterization of ICAM-1 biophore to design cytoadherence blocking peptides. *Journal of molecular graphics & modelling* **57**, 27-35, doi:10.1016/j.jmgm.2015.01.004 (2015).

3 Trott, O. & Olson, A. J. AutoDock Vina: improving the speed and accuracy of docking with a new scoring function, efficient optimization, and multithreading. *J Comput Chem* **31**, 455-461, doi:10.1002/jcc.21334 (2010).

4 Wishart, D. S. *et al.* DrugBank: a knowledgebase for drugs, drug actions and drug targets. *Nucleic Acids Res* **36**, D901-D906, doi:10.1093/nar/gkm958 (2008).

5 McRae, M. *et al.* Plasma Bile Acid Concentrations in Patients with Human Immunodeficiency Virus Infection Receiving Protease Inhibitor Therapy: Possible Implications for Hepatotoxicity. *Pharmacotherapy: The Journal of Human Pharmacology and Drug Therapy* **30**, 17-24, doi:10.1592/phco.30.1.17 (2010).

6 Mertens-Talcott, S. U., Jilma-Stohlawetz, P., Rios, J., Hingorani, L. & Derendorf, H. Absorption, Metabolism, and Antioxidant Effects of Pomegranate (Punica granatum L.) Polyphenols after Ingestion of a Standardized Extract in Healthy Human Volunteers. *Journal of Agricultural and Food Chemistry* **54**, 8956-8961, doi:10.1021/jf061674h (2006).

7 Ditscheid, B. *et al.* Effect of L-methionine supplementation on plasma homocysteine and other free amino acids: a placebo-controlled double-blind cross-over study. *European Journal of Clinical Nutrition* **59**, 768-775, doi:10.1038/sj.ejcn.1602138 (2005).
